# Additive Nanosecond Laser-Induced Forward Transfer of High Antibacterial Metal Nanoparticle Dose onto Foodborne Bacterial Biofilms

**DOI:** 10.3390/mi13122170

**Published:** 2022-12-08

**Authors:** Alena Nastulyavichus, Liliana Khaertdinova, Eteri Tolordava, Yulia Yushina, Andrey Ionin, Anastasia Semenova, Sergey Kudryashov

**Affiliations:** 1 Lebedev Physical Institute, 119991 Moscow, Russia; 2 N.F. Gamaleya Federal Research Center of Epidemiology and Microbiology, 123098 Moscow, Russia; 3 Federal State Budgetary Scientific Institution “Federal Scientific Center for Food Systems named after V.M. Gorbatov” Russian Academy of Sciences, 109316 Moscow, Russia

**Keywords:** additive laser-induced forward transfer, antibacterial nanoparticles, food bacterial films, bactericidal effect

## Abstract

Additive laser-induced forward transfer (LIFT) of metal bactericidal nanoparticles from a polymer substrate directly onto food bacterial biofilms has demonstrated its unprecedented efficiency in combating pathogenic microorganisms. Here, a comprehensive study of laser fluence, metal (gold, silver and copper) film thickness, and the transfer distance effects on the antibacterial activity regarding biofilms of Gram-negative and Gram-positive food bacteria (*Staphylococcus aureus*, *Pseudomonas aeruginosa*, *Escherichia coli*, *Listeria monocytogenes*, *Salmonella* spp.) indicated the optimal operation regimes of the versatile modality. LIFT-induced nanoparticle penetration into a biofilm was studied by energy-dispersion X-ray spectroscopy, which demonstrated that nanoparticles remained predominantly on the surface of the biofilm.

## 1. Introduction

To date, the problem of antibiotic resistance is extremely relevant throughout the world [1]. The global pandemic has made its own adjustments, and recent studies have revealed concern with regard to future rising bacterial resistance to antibiotics [2]. Pathogenic microorganisms achieve the greatest resistance due to the fact that most bacteria exist in nature not in the form of free-floating cells, but in the form of specifically organized biofilms, which are very ubiquitous in nature. The formation of biofilms has been observed by most bacteria in natural, clinical, and industrial conditions (e.g., under fluidity conditions at the boundary of two media phases (liquid–liquid, liquid–air, etc.)). Biofilms are found on solid substrates immersed in an aqueous solution and can also form floating mats on liquid surfaces. Surprisingly, the bacteria themselves make up only 5–35% of the mass of the biofilm, while the rest is the extracellular polymer matrix. The stability factor of biofilms is the mucopolymer layer, which is produced immediately after adhesion, and includes lipopolysaccharides, proteoglycans, glycoproteins, and endopolysaccharides, similar to the substance of the cell wall, glycocalyx, and bacterial capsules. An example of the protective function of a polymer film is the survival of Salmonella during chlorination, which proves the resistance of a bio-film to this method of disinfection. Bacterial biofilms impact humanity as lots of infections and diseases [3], being responsible for chronic infections, pneumonia, otitis, and arteriosclerosis [4], not to mention indwelling catheters as well as other foreign bodies [5].

Bacteria can be resistant to antibiotics due to various reasons, which could be cells in the stationary phase within the biofilm that reduce the action of antibiotics because they target biological processes. Bacteria are also able to build penetration barriers [5].

Aside from medicine, antimicrobial agents are also a necessity for water disinfection [6], food packaging [7,8], and the textile industry [1]. In the food industry, there are very favorable conditions for the formation of biofilms (presence of water, suitable surfaces for attachment, sufficient nutrients) [9]. Floor and wall surfaces can be indirect sources of bacterial contamination, which can be transferred to the product through air, personnel, and the cleaning system [10].

In the past years, foodborne pathogens have severely impacted public health and are usually underestimated because of the difficulties in establishing the cause of the disease. According to the World Health Organization (WHO) 600 million fall ill due to contaminated food and 420,000 die every year [11]. For the food industry, it is vital to have bacteria under control, and it is important to not only maintain the existing ways, but also search for new ones, since well-known pathogens may adapt, and the emerging pathogens may be already resistant to what is currently in use [12]. *Salmonella* and *E. coli* are among the most common foodborne pathogens that affect millions of people annually—frequently with severe or mortal outcomes. On the other hand, *Listeria*, despite rarely occurring, affects newborns and the elderly, sometimes with fatal health consequences [13].

Various methods are known to prevent the formation of biofilms in the food industry including their complete destruction. Mechanical processing (brushing) with the use of magnetic and pulsed electric fields are physical methods. Chemical methods include treatment with disinfectants such as chlorine, hydrogen peroxide, ozone, and essential oils [14,15,16]. In addition to these methods, there are also biological ones that use enzymes [17]. However, they are sensitive to high pressure and temperature. In addition to the fact that bacteria are constantly acquiring resistance to existing antibacterial agents, when they are in bacterial biofilms, the concentrations used are up to 1000 times higher. Therefore, the search for effective methods of combating bacterial biofilms, both with the formed ones and at the stage of their development, is currently relevant.

Recently, nanomaterials have been widely used to combat bacterial contamination. Compared with organic antibacterial agents, inorganic materials with antibacterial properties, for example, metal oxides, have good stability at high temperatures and pressures [18,19]. Among them, antibacterial nanoparticles are of particular interest. Magnetic nanoparticles are very attractive for biomedicine, being manipulated by an external magnetic field gradient for magnetic resonance imaging contrast enhancement, hyperthermia treatments, and drug delivery [20,21]. Other metal nanoparticles have great potential and are superior to conventional antibiotics due to their mechanisms of action [22,23]. Silver and zinc oxide are already used for water purification, packaging materials [24], and as anti-bacterial sprays [25]. It is known that cooper and silver oxide NPs have excellent antimicrobial properties against *E. coli*, *S. aureus*, *P. aeruginosa*, etc. [26]. There are a number of works devoted to antibacterial treatment with nanoparticles to combat planktonic culture [27,28,29] and prevent biofilm formation [30,31,32]. In the work [33], the efficiency of titanium dioxide nanoparticles against biofilms of several strains of *C. albicans* was studied, and the use of these nanoparticles to coat materials was suggested. Silver nanoparticles were successfully used to combat bacterial biofilms (*P. aeruginosa*, *Escherichia coli*, and *S. aureus*) [25,34]. Copper oxide nanoparticles have been effectively used against both Gram-positive and Gram-negative bacteria [35,36]. In addition, their efficiency has been shown to prevent the formation of new biofilms and combat already formed ones [37]. Magnesium oxide nanoparticles are also quite often used as antibacterial agents, exhibiting a significant decrease in the biomass of mature biofilm [38]. This significant effect was achieved by employing a sufficiently high concentration of nanoparticles (100–1200 µg/mL) [39].

Several mechanisms of bacterial death as a result of interaction with nanoparticles are known to date. According to the Ostwald–Freundlich equation, nanoscale particles are more susceptible to ion release due to their larger surface area compared to bulk matter, which leads to translocation and particle internalization into the cells, and later, the disruption of DNA replication [40]. In addition, it is known to change the permeability and dissipation of the proton motive force due to the generation of reactive oxygen species (ROS) from the metal nanoparticles and ions, which lead to oxidative damage to cellular structures [41], and the accumulation and dissolution of NPs in the bacterial membrane [42,43]. Nanoparticles could also interfere with microbial signal transduction: it is impacted by potentially dephosphorylating the tyrosine residuals over the peptide substrates, which might cause cell apoptosis and the inhibition of cell propagation [44].

Different methods are used for nanoparticle production. In particular, nanoparticles obtained by a method of laser ablation have a number of advantages. This method makes it possible to obtain chemically pure products, while it is characterized by the simplicity of the process control and high deposition rate [45,46]. In this work, the laser induced transfer (LIFT) method was used to obtain nanoparticles. This method is widely used for additive micropatterning [47,48,49] and has demonstrated its applicability to different metals and simple dielectric materials such as metal oxides [48,49]. LIFT-printed materials now include deoxyribonucleic acid (DNA) and other biomaterials [49]. In addition, this method is used for the production of biosensors.

In this work, we investigated the efficiency of the LIFT method for its application for the antibacterial treatment of biofilms of various Gram-positive and Gram-negative food bacteria. The effect of the concentration of the nanoparticles and the laser fluence was studied.

## 2. Materials and Methods

### 2.1. Materials and Bacterial Strains

The following bacteria were examined in the study: Gram-positive (*S. aureus*, *L. monocytogenes*) and Gram-negative (*P. aeruginosa*, *E. coli*, *Salmonella* spp.) food cultures.

To grow bacterial biofilms, the overnight broth culture was diluted 100 times in fresh sterile lysogeny broth, transferred to sterile dishes (Petri dish, microtiter plate for 12 holes, etc.), where pre-cut and sterilized silica glasses (1 cm × 1 cm) were arranged. After 18–24 h, the glasses were transferred to sterile dishes for the experiment. Biofilms were not washed or fixed. The number of samples in one experiment was three, and the number of repetitions was at least three. After transferring metal nanoparticles to the biofilms (on both sides of the glass), the test samples were placed in tubes with the physiological raster and shaken intensively for 10 min. Then, titration was carried out and bacteria were sown on the solid nutrient medium and the amount of colony forming units (CFU) was determined by the standard microbiological colony count method. As the control, we used bacterial biofilms untreated by nanoparticles.

### 2.2. Nanoparticles by the Method LIFT

**Figure 1:**
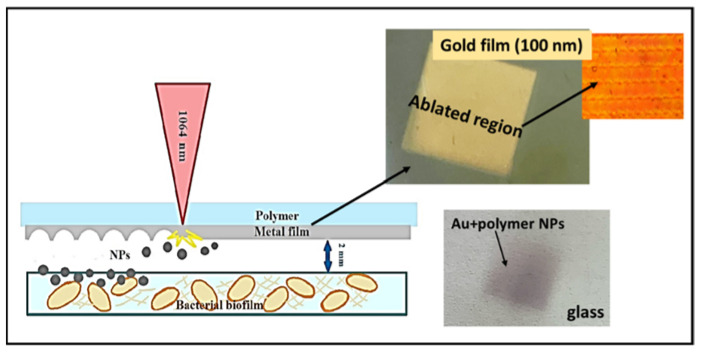
Experimental schematic; images of the ablated metal film region on the donor polymer substrate and the imprint of the transferred gold nanoparticles on the acceptor glass substrate.

For the laser-induced forward transfer of NPs on the biofilms, we used silver (Ag), gold (Au), and copper (Cu) films with thicknesses of 25, 40, 70, and 100 nm, which were obtained using the magnetron sputtering method in an argon atmosphere onto 0.5-mm thick donor polymer (polyethylene terephthalate) substrates. Film thicknesses were measured by a scanning probe microscope Certus Standard V (NanoScan Technology, Dolgoprudny, Russia). A distance ≈2 mm was set between the metal films and the glass substrates with the biofilms for the laser transfer of NPs by the Yb^3+^ nanosecond fiber laser (HTF MARK, OKB “BULAT”, Zelenograd, Russia; wavelength: 1064 nm, pulse width at half-height: 100 ns). The laser beam was focused onto the metal films using a f-theta objective lens with a focal length of 160 mm into a spot with a 1/e diameter of 50 μm. Several laser pulse energies were used in the work: 0.1, 0.2, 0.3 mJ, the pulse repetition rate was constant and equal to 20 kHz, and the scanning speed was 1500 mm/s (Figure 1). The area of 1 cm × 1 cm (biofilm sample size) was selected on the metal film, and upon laser scanning, nanoparticles were transferred to the biofilms on both glass sides.

As an example, Figure 1 demonstrates the region after laser scanning on the 100-nm thick gold film and the corresponding transferred gold nanoparticles on the glass surface.

### 2.3. Sample Characterization

Surface topography and chemical composition of the biofilms and transferred nanoparticles were characterized by their deposition onto a Si wafer, using a scanning electron microscope (SEM, TESCAN VEGA, TESCAN, Brno, Czech Republic), equipped by an energy-dispersion X-ray spectroscopy (EDX) module AZTEC (Oxford Instruments, High Wycombe, UK) for chemical microanalysis.

## 3. Results and Discussions

### 3.1. Characterization of LIFT Parameters

**Figure 2:**
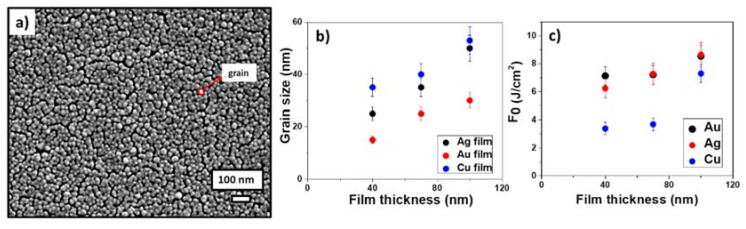
SEM visualization of the 100-nm thick Au film on the donor polymer substrate (**a**); grain dependence on the film thickness for silver, copper and gold films (**b**); and dependence of the LIFT threshold fluence (F_0_) on the metal film thickness (**c**).

The structure of the deposited metal films was visualized by SEM. Figure 2a demonstrates such a nanograin structure of the 100-nm thick gold film, with the grain size increasing versus the silver, copper, and gold film thicknesses (Figure 2b).

The corresponding LIFT (ablation) thresholds were measured for all metal films. The threshold fluences increased with the increasing film thickness for all of these metals; moreover, for the gold films, the ablation thresholds were higher and approached ≈8.6 J/cm^2^ (Figure 2c).

The monotonous thickness-dependent growth of the LIFT thresholds could be related to the increasing energy deposition depth for the thicker silver films with their high thermal conductivity [50]. For example, in [51], this effect for copper films was associated with conduction electron scattering by lattice impurities and defects in the films and on the film surfaces. Additionally, this can largely be attributed to the increasing reflectivity with the increasing film thickness. In this work, the laser transfer of nanoparticles was carried out above the ablation threshold, which made it possible to carry out the entire film completely. In addition, since the ablation threshold for gold film was higher than for the copper and silver films, this means that less material will be removed at the same fluence for gold film. This fact does not affect the antibacterial properties, since previous experiments with different energy densities for gold films did not reveal a difference in the results.

### 3.2. Optimization of LIFT Efficiency and Nanoparticle Sizes

**Figure 3:**
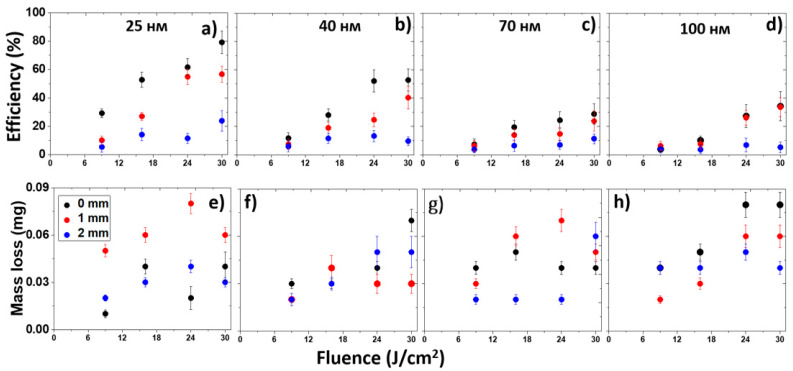
Dependence of the laser transfer efficiency evaluated from the optical density at 380 nm (**a**–**d**) and mass loss (**e**–**h**) for different distances between the target and the glass substrate on the laser fluence.

The transfer efficiency was investigated for the gold films with the thicknesses of 25, 40, 70, and 100 nm. For this purpose, during the laser transfer of nanoparticles from these films, the laser fluence was varied (9–30 J/cm^2^)) and the distance between the donor metal film and acceptor glass substrate for nanoparticle transfer was varied in the range of 0–2 mm (Figure 3). Furthermore, for the acceptor glasses with the transferred nanoparticles and initial films, the transmission spectra were recorded to acquire their optical density in the spectral range of the corresponding interband transitions in gold (≈380 nm). Based on these data, the efficiency of laser transfer was calculated depending on the fluence, film thickness, and distance between the film and glass. To do this, the ratio of the optical density of the film after laser treatment to the optical density of the untreated (original) film was taken and recalculated as a percentage. It turned out that with an increase in the film thickness, the transfer efficiency decreased and did not reach more than 40%. For comparison, the laser transfer efficiency for the 25-nm thick film reached 80%. As expected, the lowest efficiency corresponded to the largest distance of 2 mm between the film and the glass, being in the range from 3 to 25%. The largest mass deposited on the glass as a result of laser transfer corresponded to the thickest film and reached 90 μg. In general, the weight was approximately in the same range for all of these film thicknesses. Saturation was observed in the region of 16–24 J/cm^2^, accompanied by a decrease in the transferred mass, with the exception of the thickest film for the distances of 0 and 1 mm. In addition, within the error bars, an increase in mass was observed for the films with 70- and 40-nm thicknesses at the smallest distance.

**Figure 4:**
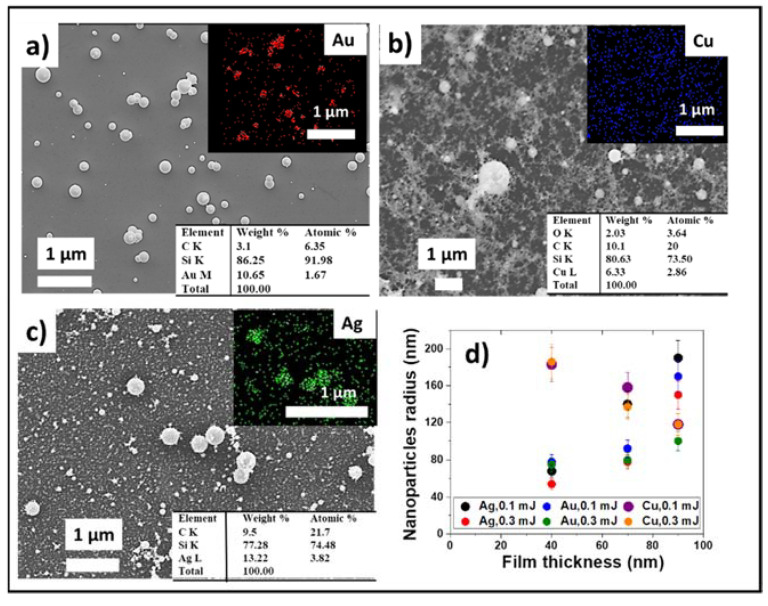
The SEM and EDX characterization of the transferred nanoparticles (**a**) Au, (**b**) Cu, (**c**) Ag; dependence of the particle sizes on the laser pulse energy and film thickness (**d**).

Nanoparticles obtained as a result of the laser transfer onto the test silicon wafer from the metal film deposited on the donor polymer substrate were examined by SEM (Figure 4). Nanoparticles of silver and copper had a fluffy carbonaceous shell (coated with fluffy PET), apparently associated with the polymer [52]. This was not observed on gold nanoparticles, and the presence of a carbon shell on the surface of the nanoparticles may be the reason for their antibacterial activity.

The particle sizes were measured by the dynamic light scattering method. Figure 4d shows the dependence of the nanoparticle size (specifically, average hydrodynamic radius) on the film thickness for the silver, gold, and copper obtained for two laser pulse energies. In general, the particle size ranged from 50 to 180 nm. With increasing energy, a decrease in nanoparticle size was observed [53]. With the increasing laser pulse energy, more defects were produced in the films and the size of the particles also decreased. In addition, small nanoparticles can be formed due to the secondary interaction of laser radiation with nanoparticles [54]. With the increase in the film thickness, the increase in the particle size was observed. For the gold films, the particle size was slightly smaller than for silver. This may be due to the fact that a carbon cap was observed from above on the silver and copper nanoparticles. The copper nanoparticles were the exception, as the average particle size decreased with the increasing film thickness.

### 3.3. Antibacterial LIFT Action on Biofilms and Its Investigation

**Figure 5:**
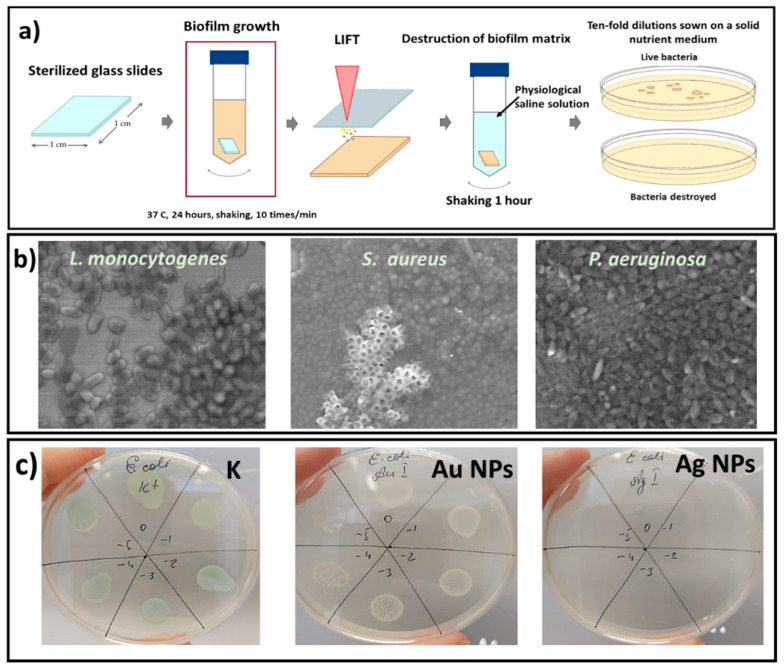
Scheme of the antibacterial investigations (**a**). SEM visualization of the bacterial biofilms (**b**). Antibacterial effect of gold and silver nanoparticles on the biofilm of *E. coli* (**c**). K is the control sample with the bacterial biofilm not treated with nanoparticles.

**Figure 6:**
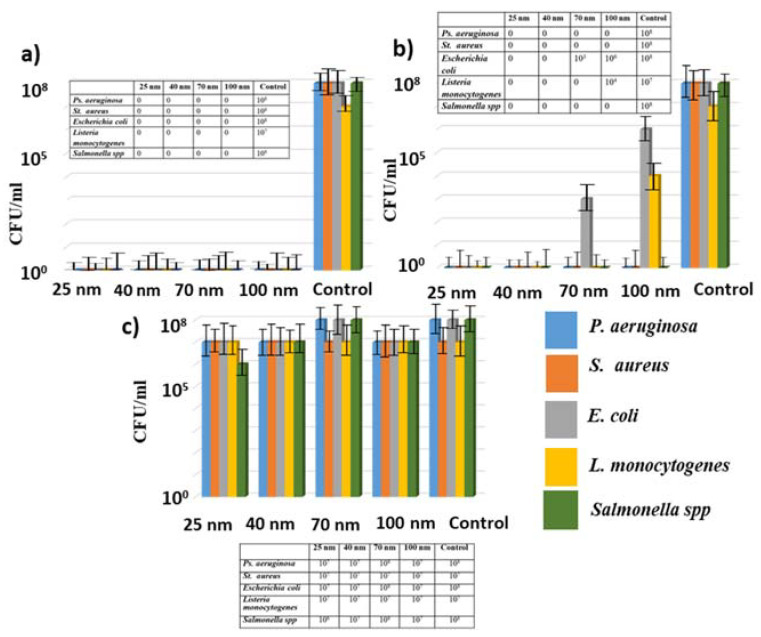
Antibacterial effect (in CFU/mL) of Cu NPs (**a**); Ag NPs (**b**); Au NPs (**c**) transferred from metal films of various thicknesses on biofilms of clinical isolates; inserts: tabular data.

In this work, the daily bacterial biofilms statically grown on the glass substrates were used in this research. In vitro studies were performed on the biofilms of Gram-positive (*S. aureus*, *L. monocytogenes*) and Gram-negative (*P. aeruginosa*, *E. coli*, *Salmonella* spp.) bacteria. Figure 5b shows the images of biofilms of some of the bacteria used in this work. Previously, the effect of silver, copper, and gold nanoparticles transferred onto *S. aureus* and *P. aeruginosa* biofilms from the 100-nm thick metal films was shown. In this work, we expanded the number of bacteria strains, and also investigated the effect of the film thickness and the laser fluence in order to identify the optimal LIFT parameters. After the laser-induced nanoparticle transfer at the fixed fluence of 16 J/cm^2^, the glass substrates with the biofilms were transferred to tubes with a saline solution of DNAse and vigorously shaken on a shaker for 1 h (Figure 5a). Under this influence, the biofilm matrix was destroyed, and the bacterial cells remained unharmed. Then, the resulting suspension was titrated by a standard microbiological method, 10-fold dilutions were prepared and sown on a solid nutrient medium to determine the corresponding CFU values (colony-forming unit) (Figure 5 and Figure 6). Figure 5c shows a few images of one typical experiment to determine the antibacterial properties of the metal nanoparticles. The studies used an adapted version of the classical method of 10-fold titration of microorganisms—microtitering. This method allows researchers to save not only on materials, but also on work time. Titration was carried out in microtiter plates. To calculate the CFU, Petri dishes were divided into six sectors and, according to the titration values, a culture in the amount of 30 µL was introduced. The obtained results were recalculated to CFU/mL.

Similar conditions were used for all of the bacterial biofilms. The antibacterial effect of the nanoparticles was investigated by comparing samples treated with the nanoparticle samples and untreated ones (biofilm grown control). Previously, it was shown in [52] that laser radiation itself does not affect the bacterial biofilms.

Studies have shown that nanoparticles of silver and copper have pronounced antibacterial properties. The obtained results demonstrated the almost complete suppression of the microorganism growth, which was manifested by the significant decrease in the number of CFU of the bacteria studied (Figure 6a,b).

At the same time, the gold nanoparticles did not affect the viability of the studied microorganisms and the number of microorganisms remained almost the same as in the control samples (Figure 6c). No bactericidal effect was observed upon the transfer of gold nanoparticles and direct exposure to the laser through the polymer donor substrate without any metal film. These results apparently show that the bactericidal activity is not related to laser exposure and the temperature of the metal nanoparticles.

**Figure 7:**
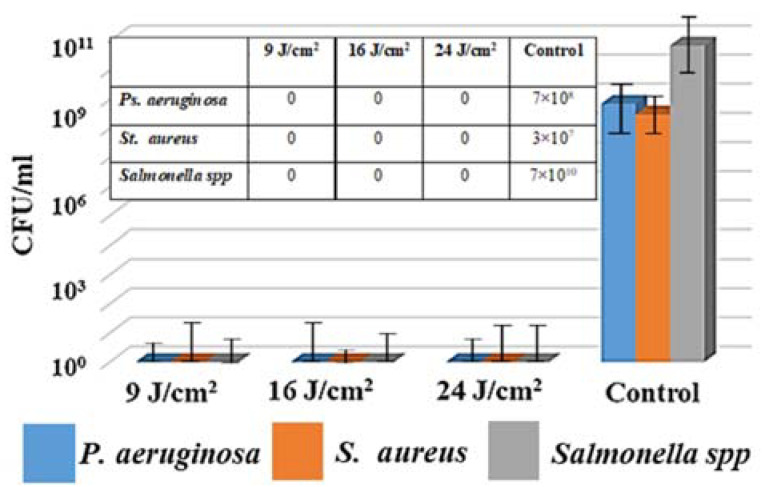
Antibacterial effect (in CFU/mL) of Ag NPs transferred at various laser fluences from silver metal films on biofilms of clinical isolates; inserts: tabular data.

We also studied the effect of the laser fluence on the transfer of the nanoparticles onto the bacterial biofilms. As an example, Figure 7 demonstrates the results of microtitering for silver nanoparticles, transferred at the different fluences. As can be seen, the decrease or increase in laser fluence did not affect the survival of bacteria.

The possible mechanism, responsible for the bacteria death, is the presence of reactive oxygen species (ROS) including the singlet oxygen on the nanoparticle surfaces. It is known that oxidative stress is responsible for the damage of cellular proteins, DNA, and lipids [52]. Ions released from the metal oxide can penetrate into a cell membrane and then interact with functional groups of proteins and nucleic acids (mercapto, carboxyl, and amino groups). Such interaction can change the cell structure, affect the intrinsic physiological processes, and lead to the death of bacteria [55].

**Figure 8:**
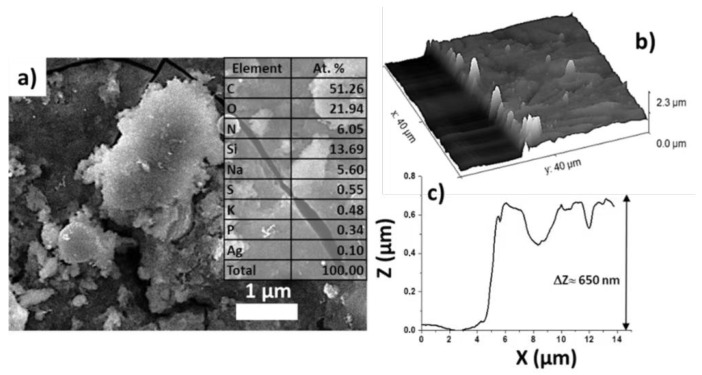
SEM visualization of *Salmonella* bacteria with transferred Ag NPs (**a**); 3D map of *Salmonella* biofilm with Ag NPs (**b**); biofilm–glass boundary profile (**c**).

Using the biofilm of the Gram-negative bacteria *Salmonella* with the transferred silver nanoparticles as an example (Figure 8a), its thickness was measured by SEM. For this purpose, a cut was made on the surface of the biofilm with a needle and SEM scanning was carried out at the biofilm–glass interface (Figure 8b). As a result, based on the presented profile (Figure 8c), it was shown that the total thickness was about 650 nm.

**Figure 9:**
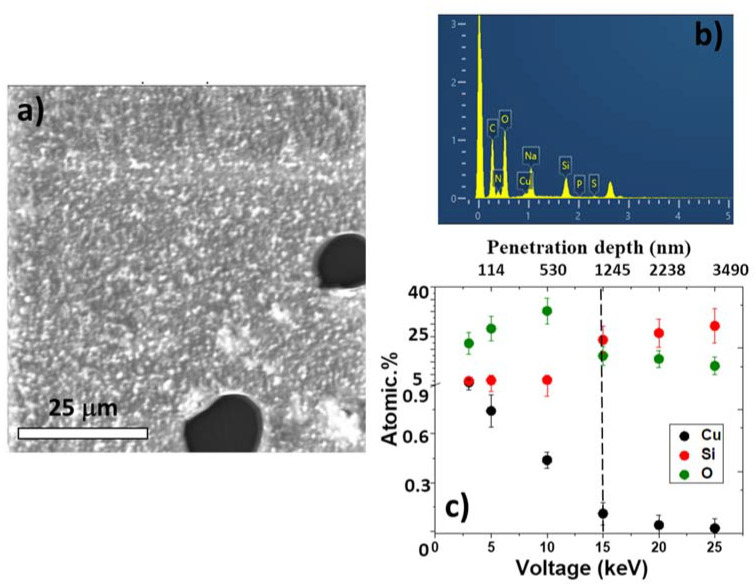
SEM visualization of the *Salmonella* bacterial biofilm with transferred copper NPs (**a**); its EDX spectrum (5 keV) (**b**). In-depth EDX distribution of oxygen (biofilm component), silicon (substrate component), and copper (component of transferred NPs), derived from their voltage dependences (**c**). The vertical dashed line indicates the Si substrate position and the rear side of the biofilm.

In addition, a characteristic penetration depth of copper nanoparticles into the LIFT-treated *Salmonella* biofilm (Figure 9a) was carried out by EDX. For this purpose, the EDX spectrum of the biofilm surface with the Cu particles was acquired at different accelerating voltages (3, 5, 10, 15, 20, 25 keV) (Figure 9b). For these accelerating voltages, the corresponding electron penetration/X-ray escape depths were different (top axis in Figure 9c). As a result, the atomic contents of some characteristic elements representing the biofilm, Si substrate, and Cu NPs could be determined by EDX analysis as a function of depth inside the biofilm, and plotted versus the acceleration voltage (penetration depth).

As can be seen from Figure 9c, the sharp decrease in oxygen content in the point, corresponding to 15 keV, and the silicon content increase in this region indicate that the thickness of the biofilm was about 1.2 μm. Simultaneously, the copper content (in at. %) rapidly decreased into the biofilm, which indicates that most of the nanoparticles were located mainly on the surface of the biofilm, providing their bactericidal effect on the entire biofilm indirectly.

## 4. Conclusions

In this work, additive laser-induced forward transfer of antibacterial metal nanoparticles onto biofilms of Gram-positive and Gram-negative food bacteria was studied in detail in terms of the basic regimes by varying the laser fluence, metal type and film thickness, and nanoparticle transfer distance. It has been shown that it is sufficient to use the minimal thickness (25 nm) of the metal films (small concentration of nanoparticles) and the minimal laser fluence of 9 J/cm^2^ to inactivate the biofilm. Compared to the nanoparticle-based surface coatings, nanoparticles of silver and copper transferred onto a biofilm have proven their excellent antibacterial activities regarding chemically-inert gold by demonstrating almost complete suppression of the food bacterial biofilms. The EDX method was used to estimate the penetration depth of LIFT-transferred nanoparticles inside the biofilm to enlighten their bactericidal effect. Thus, it was shown that nanoparticles did not penetrate into the entire biofilm and thus acted on it indirectly from outside. This method allows one to quickly inactivate mature bacterial biofilms with its potential applications in medicine for the treatment of wounds as a plaster and in the food industry for the bactericidal treatment of functional surfaces.

## Data Availability

Not applicable.
